# Tunable C_4_-Symmetry-Broken Metasurfaces Based on Phase Transition of Vanadium Dioxide (VO_2_)

**DOI:** 10.3390/ma17061293

**Published:** 2024-03-11

**Authors:** Yuting Zhang, Xiaoyuan Hao, Xueguang Lu, Meng Liu, Wanxia Huang, Cheng Zhang, Wei Huang, Yi Xu, Wentao Zhang

**Affiliations:** 1 Guangxi Key Laboratory of Optoelectronic Information Processing, School of Optoelectronic Engineering, Guilin University of Electronic Technology, Guilin 541004, China; lzytch@guet.edu.cn (Y.Z.); haoxiaoxiao1996@163.com (X.H.); weihuang@guet.edu.cn (W.H.); yi_xu@guet.edu.cn (Y.X.); 2 College of Materials Science and Engineering, Sichuan University, Chengdu 610065, China; luxueguang@stu.scu.edu.cn (X.L.); huangwanxia@scu.edu.cn (W.H.); 3 College of Electronic and Information Engineering, Shandong University of Science and Technology, Qingdao 266590, China; 4 College of Science, Wuhan University of Technology, Wuhan 430070, China; czhang2020@whut.edu.cn

**Keywords:** four-fold rotation (C_4_) symmetry, electromagnetically induced transparency (EIT), metasurface, vanadium dioxide (VO_2_), chirality

## Abstract

Coupling is a ubiquitous phenomenon observed in various systems, which profoundly alters the original oscillation state of resonant systems and leads to the unique optical properties of metasurfaces. In this study, we introduce a terahertz (THz) tunable coupling metasurface characterized by a four-fold rotation (C_4_) symmetry-breaking structural array achieved through the incorporation of vanadium dioxide (VO_2_). This disruption of the C_4_ symmetry results in dynamically controlled electromagnetic interactions and couplings between excitation modes. The coupling between new resonant modes modifies the peak of electromagnetic-induced transparency (EIT) within the C_4_ symmetric metasurfaces, simulating the mutual interference process between modes. Additionally, breaking the C_4_ symmetry enhances the mirror asymmetry, and imparts distinct chiral properties in the far-field during the experimental process. This research demonstrates promising applications in diverse fields, including biological monitoring, light modulation, sensing, and nonlinear enhancement.

## 1. Introduction

Metamaterials are artificially designed arrays of sub-wavelength microstructures capable of manipulating electromagnetic waves arbitrarily through the free design of microstructures. Specifically, metamaterials can achieve anomalous and novel electromagnetic phenomena not attainable in traditional materials, sparking extensive research interest in electromagnetic manipulation [1,2]. Metasurfaces, on the other hand, are two-dimensional metamaterials comprised of subwavelength periodic arrays with ultra-thin thickness, offering advantages such as easy processing, low loss, and an ultra-compact configuration [3,4]. As a ubiquitous phenomenon observed in various systems, coupling phenomenon profoundly alters the original oscillation state of resonant systems and leads to unique optical properties [5,6]. In the realm of metasurfaces, coupling effects have been demonstrated to significantly modify the behavior of resonant systems, resulting in enhanced optical characteristics. For instance, coupling has been instrumental in facilitating well-known physical phenomena such as electromagnetically induced transparency (EIT) [7,8], Fano resonance [9,10], and so forth., In particular, EIT manifests as a narrow transparency window within the original absorption spectrum. Owing to its promising applications, EIT has garnered significant attention in the optical field, including enhanced transmission windows and strong dispersion characteristics. However, previous EIT devices have displayed fixed functionalities once the proposed metasurfaces were fabricated, constraining their broadened applications. In this study, we have introduced dynamically controlled terahertz (THz) metasurfaces based on elementary cells with tunable optical properties to overcome these limitations [11,12]. Previous studies have incorporated phase change materials into metasurfaces to achieve dynamic functionality. Among the commonly used phase change materials, vanadium dioxide (VO_2_) is a more suitable candidate due to the relatively low excitation threshold of external stimulus [13,14]. Particularly, VO_2_ undergoes a reversible insulator-to-metal transition (IMT) around room temperature (68 °C), making it an ideal choice for developing dynamically controlled energy efficient devices. Therefore, the metal–insulator phase transitions of VO_2_ in metasurfaces have become a research hotspot in the design dynamically controlled THz metasurfaces.

In 2019, Xingbo Liu et al. proposed temperature-controlled THz holography based on the phase transition of VO_2_ [15]. Through the deposition of VO_2_ under C-shaped split-ring resonators, the conductivity of VO_2_ can be dynamically modulated by adjusting the operating temperature. This leads to a modification of the resonant properties of the split-ring resonators (SRRs) and the reconstruction of the holographic image. This innovative approach provides a dynamic and tunable method for controlling holography, showcasing potential practical applications in fields such as display technology and data storage. In 2013, Xueqian Zhang et al. introduced a polarization-independent EIT THz metasurface. The proposed metasurface unit cell comprises a cross and four identical SRRs positioned at equal distances within the four quadrants formed by the cross. This configuration exhibits four-fold rotational symmetry (C_4_ symmetry) and manifests EIT electromagnetic properties through the coupling effect of four distinct modes [16]. C_4_ symmetry structures possess rotational symmetry that cannot be superimposed with their mirror image through simple rotation or translation. As a result, C_4_ symmetry structures are frequently employed in the design of chiral metasurfaces, such as 4-U-SRRs [15], corner-stacked rods [17], conjugated gamma resonator pairs [18], among others. Chiral metasurfaces have been demonstrated to have wide-ranging applications, including biological monitoring [19], nonlinear enhancement [20], circular polarizers [21], and so on [22,23]. 

Previous studies (Xueqian Zhang et al.) have demonstrated that the EIT structure conforms to C_4_ symmetry and is characterized by the coupling of four distinct modes [16]. This approach acknowledges that EIT typically arises from the coupling between bright and dark modes. The bright mode is stimulated by incident radiation, whereas the dark mode is excited by the near-field coupling originating from the resonator of the bright mode. Utilizing this classical C_4_ symmetric structure as a foundation, we exert control over the coupling in the structure by incorporating phase change materials.

In this research, we experimentally demonstrated a C_4_ symmetry-broken EIT structure by designing VO_2_ split-ring resonators (SRRs) or rings beneath the surface metal SRRs. The original C_4_ symmetry EIT structure consists of a cross and four identical SRRs, exhibiting four distinct modes: super-radiance mode (SM), superdark mode (DM), subradiance mode (sM), and subdark mode (dM). In our design, VO_2_ rings were inserted under one, two, three, and four SRRs, respectively, with their conductivity can be actively controlled through temperature regulation. By tailoring the conductivity of VO_2_, the C_4_ symmetry of the EIT structure is disrupted, resulting in modifications to the sM and dM structures. Consequently, the internal coupling modes of the C_4_ symmetry EIT structure were manipulated accordingly. In the following sections, we will delve into the impact of VO_2_ on inter-mode coupling in the EIT structure through detailed analyses of experimental and simulation results for VO_2_ rings designed beneath one, two, three, and four surface metal SRRs, respectively.

## 2. Device Design and Simulation Results

**Figure 1:**
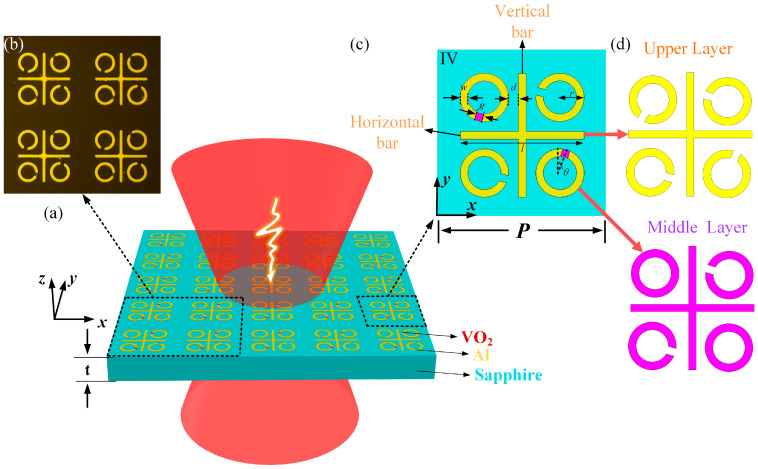
(**a**) The schematic diagram of the proposed C_4_-symmetry-broken EIT metastructure and the unit cell. (**b**) The optical microscopic image of the fabricated sample. (**c**) The unit structure diagram of the third model, and the dimensions are shown in the illustration. (**d**) Separate display of two-layer structure.

The proposed tunable C_4_-symmetry-broken metasurfaces are structured in three layers, as illustrated in Figure 1. The upper layer comprises aluminum (Al) metallic crosses and split-ring resonators (SRRs). In the middle layer, VO_2_ crosses, SRRs, and rings are arranged. The SRRs are arranged to conform to C_4_ symmetry and are distributed in four quadrants. The linewidths of VO_2_ crosses, SRRs, and rings match that of the metallic crosses and SRRs. The lower layer consists of a C-cut sapphire substrate. 

Various combinations of VO_2_ rings and SRRs have been used to design six different structures: (I) All VO_2_ SRRs are placed in the four quadrants. (II) One VO_2_ ring is positioned in the first quadrant, while VO_2_ SRRs occupy the other quadrants. (III) Two VO_2_ rings are located in the first and second quadrants, with two VO_2_ SRRs in the third and fourth quadrants. (IV) Two VO_2_ rings are positioned in the second and fourth quadrants, with two VO_2_ SRRs in the first and third quadrants. (V) Three VO_2_ rings occupy the first to third quadrants, while one VO_2_ SRR is placed in the fourth quadrant. (VI) Four VO_2_ rings are distributed across all quadrants.

The geometric parameters of the proposed C_4_-symmetry-broken EIT metastructure are provided in Figure 1c. The cross length is *l* = 82 µm, and the width of all metal wires is *w* = 5 µm. The outer radius of SRRs and rings is *r* = 16 µm, and the gap width is *g* = 5 µm. The relative center distance from an SRR or ring to a cross in the four quadrants is *d* = 25 µm, respectively. The gap of the SRRs is at an angle of *θ* = 15° with respect to the adjacent bars of the cross. The period of the unit cell is *P* = 120 µm, and the substrate thickness is *t* = 640 µm. The thickness of VO_2_ layer and Al layer are both 200 nm. The conductivity of Al is set to 3.56 × 10^7^ S/m, and the relative dielectric constant of C-cut sapphire is *ε* = 9.4. The period *P* of the unit cell is much smaller than the operation wavelength.

As a phase change material, VO_2_ film undergoes a transition from monoclinic to tetragonal phases when heated from low to high temperatures. This phase change occurs rapidly around the critical temperature (TC = 68 °C), leading to significant alterations in the lattice structure. During this transition, various parameters of VO_2_ thin films undergo substantial changes, such as transmittance, absorptivity, reflectivity, conductivity, refractive index, and magnetic susceptibility. The bulk conductivity of single-crystal VO_2_ thin films can reach four orders of magnitude or higher. This conductivity change is attributed to the distortion of the VO_2_ crystal lattice at the critical temperature, which alters the periodic potential and affects the crystal’s energy band structure. The lattice distortion in VO_2_ thin films induces the splitting and movement of three-dimensional electronic states parallel to the C-axis of the tetragonal rutile phase. As a result, the Fermi level shifts from the original conduction band to the forbidden band, facilitating the transition from insulator to metal. The phase transition temperature of VO_2_, occurring at 68 °C, aligns closely with room temperature, making the electrical and optical characteristics of VO_2_ films grown on sapphire substrates abrupt. These characteristics, resembling those of crystal materials, provide VO_2_ films with broad applications in temperature sensing, optics, and intelligent electrical switches. Achieving the phase transition of VO_2_ is possible through electronic control and thermology. Under thermal control, VO_2_ can reversibly transition from an insulating state to a metallic state. In our simulations, we utilized VO_2_ conductivity σ = 200 S/m at 25 °C to replicate the transmission characteristics of the metasurface at room temperature and σ = 2 × 10^5^ S/m at 68 °C to simulate the transmission characteristics at high temperature [24,25,26,27]. 

**Figure 2:**
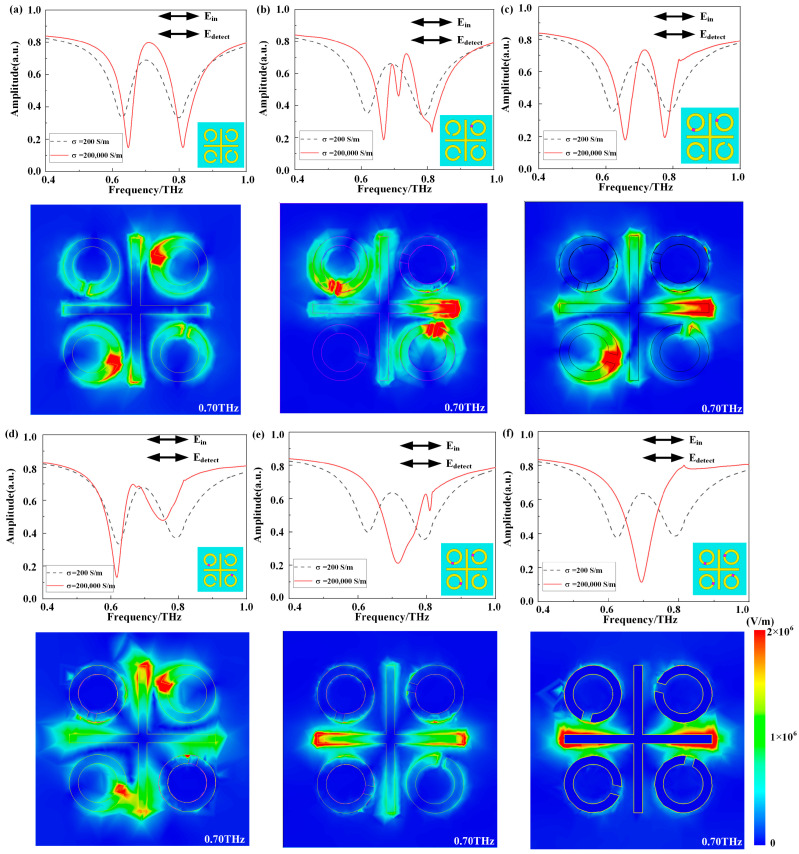
(**a**–**f**) The amplitude transmission spectra and electric field distributions for *x*-polarized waves at normal incidence are presented for six models. In the simulations, the transmission spectra are depicted with σ = 200 S/m, representing VO_2_ at room temperature, and with σ = 2 × 10^5^ S/m, representing VO_2_ over 68 °C. Furthermore, we present the surface electric field distributions corresponding to *x*-polarized waves at 0.70 THz and 68 °C.

We analyzed the internal coupling modes of the C_4_ symmetry-broken EIT structure for each model through simulation. Numerical simulations were performed using CST Microwave Studio 2019, a three-dimensional finite element electromagnetic simulation software. The boundary conditions were set as ±x and ±y directions as unit cells and ±z as open. We utilized a frequency domain solver of CST Microwave Studio 2019 for steady-state calculations. Sapphire and aluminum materials utilize default material parameters in CST Microwave Studio, while VO_2_ employs the Drude model. The grid structure was configured using a hexagonal grid. At the EIT coupling mode of 0.70 THz, Figure 2a illustrates the stimulation and coupling between SM (i.e., the horizontal bar) and DM (i.e., the vertical bar), dM (i.e., the SRRs in the first and third quadrant), and sM (i.e., the SRRs in the second and fourth quadrant), leading to the EIT phenomenon. In Figure 2b at the same coupling mode, SM (i.e., the horizontal bar) was stimulated and coupled with DM (i.e., the vertical bar), dM (i.e., the SRRs in the third quadrant), and sM (i.e., the SRRs in the second and fourth quadrant), resulting in an incomplete EIT phenomenon. The electric field simulations in Figure 2c indicate that EIT coupling primarily occurs between SM (i.e., the horizontal bar), DM (i.e., the vertical bar), dM (i.e., the SRR in the third quadrant), and sM (i.e., the SRR in the fourth quadrant), resulting in a relatively complete EIT peak due to left–right symmetry. Figure 2d depicts SM (i.e., the horizontal bar) coupled with dM (i.e., the SRRs in the first and third quadrants) and DM (i.e., the vertical bar). In Figure 2e, two resonance modes without an EIT phenomenon are observed—SM (i.e., the horizontal bar) and sM (i.e., the SRR in the fourth quadrant). At 0.70 THz, the primary resonance mode triggered by SM (i.e., the horizontal bar) produces a peak, while sM’s resonance is relatively weaker. Finally, Figure 2f confirms, through transmission spectra and electric field simulation outcomes, that only SM (i.e., the horizontal bar) is directly triggered, resulting in a resonance peak at 0.70 THz. The analysis of the transmission spectra and electric fields for the *x*-polarized incident waves highlights that the EIT phenomenon primarily relies on the coupling between SM and dM. Nevertheless, due to the non-coplanar nature of the VO_2_ and metal structures, a height difference of 200 nm arises. Consequently, the electric field simulation results still exhibit the presence of an SRR. Despite this, the metal thickness is minimal, and the electric field at the opening is weak, ensuring negligible impact on the overall results.

**Figure 3:**
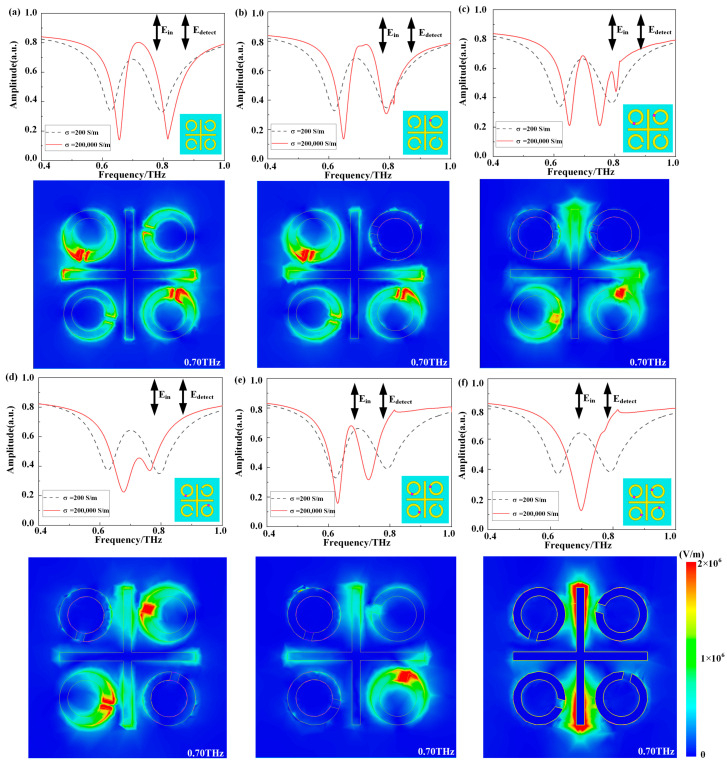
(**a**–**f**) The amplitude transmission spectra and electric field distributions of the six models for *y*-polarized waves at normal incidence are presented. The transmission spectra, representing VO_2_ at room temperature (σ = 200 S/m) and VO_2_ over 68 °C in the simulation (σ = 2 × 10^5^ S/m), respectively. Additionally, the surface electric field distributions for *y*-polarized waves at 0.70 THz over 68 °C are illustrated.

In Figure 3a at the EIT coupling mode of 0.70 THz, the vertical bar (SM) is stimulated and coupled with the horizontal bar (DM), dM (i.e., the SRRs in the second and fourth quadrant), and sM (i.e., the SRRs in the first and third quadrant), leading to the EIT phenomenon. Figure 3b shows that at the EIT coupling mode of 0.70 THz, SM (i.e., the vertical bar) is stimulated and coupled with DM (i.e., the horizontal bar), dM (i.e., the SRRs in the second and fourth quadrant), and sM (i.e., the SRRs in the third quadrant), resulting in an incomplete EIT phenomenon. In Figure 3c, the electric field simulation results indicate that EIT coupling mainly occurs between SM (i.e., the vertical bar), DM (i.e., the horizontal bar), dM (i.e., the SRR in the fourth quadrant), and sM (i.e., the SRR in the third quadrant). Due to left–right symmetry, the EIT peak is relatively complete. Figure 3d depicts SM (i.e., the vertical bar) weakly coupled with sM (i.e., the SRRs in the first and third quadrants) and DM (i.e., the horizontal bar). In Figure 3e, at the EIT coupling mode, SM (i.e., the vertical bar) is stimulated and coupled with DM (i.e., the horizontal bar) and dM (i.e., the SRR in the fourth quadrant), resulting in an incomplete EIT phenomenon. Finally, Figure 3f confirms, through the analysis of transmission spectra and electric field simulation outcomes, that only SM (i.e., the vertical bar) is directly triggered, producing a resonance peak at 0.70 THz. The analysis of the transmission spectra and electric fields for the *y*-polarized incident waves further verifies the EIT phenomenon based on the coupling between SM and dM.

## 3. Experimental Verification

The metasurfaces were primarily fabricated using semiconductor device processing technology for metal and dielectric THz metasurfaces. The process began with depositing a layer of VO_2_ on a sapphire substrate. Subsequently, a uniform layer of photoresist was applied, and an optimal pattern was obtained through exposure and immersion in a developing solution. Following this, the VO_2_ layer underwent etching using reactive ion etching (RIE) with CF_4_. Finally, the aluminum metal structure was prepared through an etching process.

During the metasurfaces’ characterization, the THz time domain spectroscopy (TDS) system was employed to assess the transmission characteristics of the sample. To analyze the sample’s transmission characteristics at high temperatures, heating was facilitated using a perforated hot plate. To ensure the completion of the phase transition in VO_2_ within the structure, the temperature of the hot plate was set to 100 °C.

**Figure 4:**
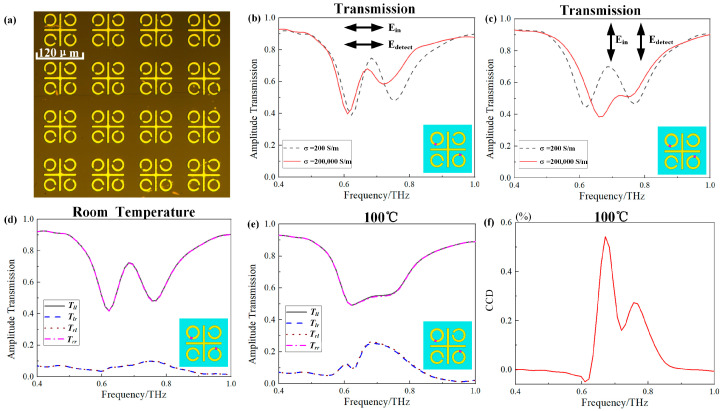
(**a**) Part of the microscopic image of the fabricated sample of the third model. (**b**,**c**) The transmission amplitude spectra with *x*-polarized and *y*-polarized incident waves in the third model experiments, respectively. (**d**,**e**) The transmission amplitude spectra of circularly polarized incident waves in the third model experiments at room temperature or 100 °C, respectively. (**f**) The experimental spectral line of CCD value. The vertical ordinate is the CCD value, which is in hundredth increments.

Figure 4a displays a partial microscopic image of the fabricated tunable C_4_-symmetry-broken metasurfaces. During the fabrication process, the samples were initially grown using the molecular beam epitaxy method. Subsequently, a 200 nm thick VO_2_ layer was deposited on a 640 µm thick C-cut sapphire substrate. To achieve the desired VO_2_ structure, a combination of deep RIE and traditional lithography technology was utilized to etch the VO_2_ layer of these devices. The metallic structures were created through the traditional lithography process, followed by deposition of a 200 nm thick aluminum film on the metallization. The microscopic image in Figure 3a indicates that both the VO_2_ structure and the metallic structure were fabricated with high quality.

To validate the accuracy of the simulation results, we conducted experimental measurements on the third and fourth models. Initially, we measured the transmission amplitudes of these models. Figure 4b,c displays the transmission amplitude spectra with the *y*-polarized and *x*-polarized incident waves of the third model at both room temperature and 100 °C. The simulation results exhibit good agreement with the experimental results. Furthermore, we measured the spectral transmission response with circular-polarized incident waves for the third model at both room temperature and 100 °C. Figure 4d,e illustrates the transmission spectra with circular-polarized incident waves at room temperature and 100 °C, respectively. We defined the vibration direction of right-handed circular polarization (RCP) as rotating clockwise along the light propagation, while the vibration direction of left-handed circular polarization (LCP) rotates counterclockwise along the light propagation. The notations *T_ll_*, *T_lr_*, *T_rl_*, and *T_rr_*, respectively, represent the transmission coefficients for LCP incidence and LCP outgoing, RCP incidence and LCP outgoing, LCP incidence and RCP outgoing, and RCP incidence and RCP outgoing. At room temperature, a prominent transmission window appears at 0.70 THz. The third model acts as an EIT device for circular-polarized incident waves at room temperature, where the transmission window is created through the interference of the coupling between the bright mode (i.e., vertical bar) and the dark mode (i.e., the SRR in the second and fourth quadrants). However, when the temperature increases to 100 °C, the VO_2_ rings in the second and fourth quadrants exhibit the resonance of the metal rings, causing the bright transmission window to vanish. As shown in Figure 4e, the co-polarized transmittance curves for circular-polarized waves are identical, but the polarization conversion transmittance curves differ. When the ambient temperature is 100 °C, due to the similarity of the two VO_2_ SRRs in the first and second quadrants to ring structures, the third model transforms into a chiral device. The primary principle of chirality lies in the severe breakdown of mirror symmetry at 100 °C. Because our designed structure consists of only a single layer, circular dichroism manifests primarily in the polarization conversion component of circular polarization. Therefore, the polarization conversion circular dichroism (CCD) value is defined by CCD = |*T_rl_*^2^–*T_lr_*^2^|. The maximum CCD value observed during the experiment in Figure 4f is 0.5%.

**Figure 5:**
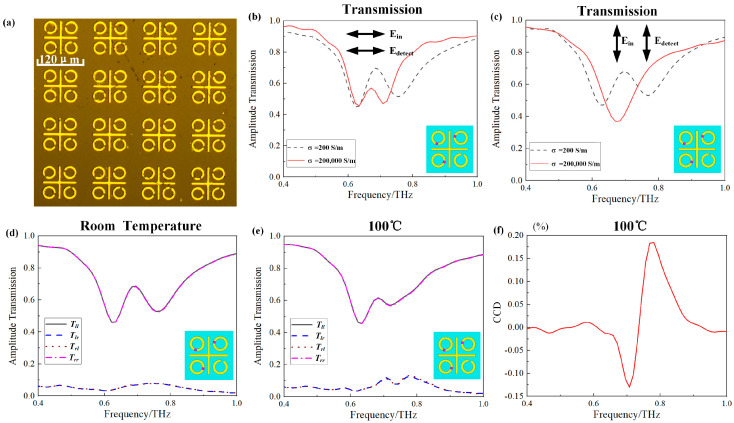
(**a**) Part of the microscopic image of the fabricated sample of the fourth model. (**b**,**c**) The transmission amplitude spectra with *x*-polarized and *y*-polarized incident waves in the fourth model experiments, respectively. (**d**,**e**) The transmission amplitude spectra of circularly polarized incident waves in the fourth model experiments at room temperature or 100 °C, respectively. (**f**) The experimental spectral line of CCD value, which is in hundredth increments.

As depicted in Figure 5a, the fabricated sample of the fourth model exhibited remarkable performance. In Figure 5b, the transmission amplitude spectra for *y*-polarized and *x*-polarized incident waves of the fourth model during the experiment at both room temperature and 100 °C align well with the simulation outcomes. Concerning the transmission spectra of linear polarization, the experimental results match those obtained from simulations. According to the experimental outcomes, the fourth model functions as an EIT device utilizing a circular-polarized incident wave at room temperature. Similar to the third model, a prominent transmission window is observed at 0.70 THz in Figure 5b. However, as the temperature rises to 100 °C, the size of the transmission window diminishes. With three VO_2_ SRRs in the first, second, and third quadrants exhibiting the characteristics of metal rings, the mirror symmetry is significantly disrupted, leading to differing polarization conversion transmittance curves. At room temperature, the fourth model closely resembles the third model, manifesting a noticeable bright EIT window. Figure 5c illustrates a transmission window at 0.70 THz for a circular-polarized incident wave. However, unlike the third model, a small bright transmission window persists in the fourth model at 100 °C, as shown in Figure 5d. This phenomenon suggests a robust electromagnetic coupling within the internal structure of the fourth model. Analysis of the model revealed that within this model, only the bright mode (i.e., the vertical bar) and the dark mode (i.e., the SRR in the fourth quadrant) exhibit direct coupling. The maximum value of CCD observed during the experiment was 0.2% at 0.79 THz, as illustrated in Figure 5f.

## 4. Conclusions

Through both experimental characterization and numerical simulation, we have demonstrated the active regulation of functional coupling in the metasurface based on a C_4_ symmetry structure comprising metallic and VO_2_ components. The responses of the EIT structure can be dynamically controlled by the external temperature, leveraging the phase transition property of VO_2_. In this study, we deliberately disrupted the C_4_ symmetry of the EIT structure by modifying the conductivity of VO_2_. We systematically analyzed how the coupling model within the EIT structure evolves as the C_4_ symmetry is gradually broken. Various design structures with approximately one, two, three, and four SRRs transitioned to circular rings, respectively, were employed to achieve this objective. The intentional breaking of the original C_4_ symmetry structure initiates a new coupling mode, giving rise to novel electromagnetic properties. This innovative structural coupling mode is pivotal for developing well-defined metasurface architectures and holds significant potential for the advancement of novel coupling properties and related coupler devices. Furthermore, the active control approach is not confined to temperature control alone, as it can also be stimulated electrically and optically. With ongoing research on C_4_ symmetry structures and rapid technological advancements, we anticipate that the potential of dynamic metasurface coupling between C_4_ symmetry structures and disrupted C_4_ symmetry structures will eventually be fully harnessed. Designs featuring disrupted C_4_ symmetry possess unprecedented potential in various applications, including slow light, biological monitoring, and nonlinear enhancement.

## Data Availability

Data are contained within the article.
